# 3D reconstruction in endonasal pituitary surgery

**DOI:** 10.1007/s11548-025-03362-9

**Published:** 2025-04-11

**Authors:** Dannielle Lee, Laurent Mennillo, Emalee Burrows, Jia-En Chen, Danyal Z. Khan, Joachim Starup-Hansen, Danail Stoyanov, Matthew J. Clarkson, Hani J. Marcus, Sophia Bano

**Affiliations:** 1 UCL Hawkes Institute, University College London, London, UK; 2 Department of Computer Science, University College London, London, UK; 3 Department of Neurosurgery, National Hospital for Neurology and Neurosurgery, London, UK; 4 Department of Medical Physics and Biomedical Engineering, University College London, London, UK

**Keywords:** 3D reconstruction, Endoscopic surgery, Learned feature detectors, Augmented reality

## Abstract

**Purpose:**

Endoscopic transsphenoidal surgery for pituitary tumors is hindered by limited visibility and maneuverability due to the narrow nasal corridor, increasing the risk of complications. To address these challenges, we present a pipeline for 3D reconstruction of the sellar anatomy from monocular endoscopic videos to enhance intraoperative visualization and navigation.

**Methods:**

Data were collected through a user study with trainee surgeons, and the procedure was conducted on 3D printed, anatomically correct phantom devices. To overcome limitations posed by the uniform, textureless surfaces of these devices, learned feature detectors and matchers were leveraged to extract meaningful information from the images. The matched features were reconstructed using COLMAP, and the resulting surfaces were evaluated using the iterative closest point algorithm against the CAD ground-truth surface of the printed phantoms.

**Results:**

Most methods resulted in accurate reconstructions with moderate variability in cases with high blur or occlusions. Average RMSE values of 0.33 mm and 0.41 mm, for the two best methods, Dense Kernelized Feature Matching and SuperPoint with LightGlue, respectively, were obtained in the surface registrations across all test sequences, with a significantly higher computation time for Dense Kernelized Feature Matching.

**Conclusion:**

The proposed pipeline was able to accurately reconstruct anatomically correct 3D models of the phantom devices, showing potential for the use of learned feature detectors and matchers in real time for AR-guided navigation in pituitary surgery.

## Introduction

Endoscopic pituitary surgery [8] is a minimally invasive procedure used to remove tumors from the pituitary gland, which is located at the base of the brain and is surrounded by critical anatomical structures, such as the carotid arteries and optic nerves. The surgery is typically performed by inserting a monocular endoscope and surgical instruments into the nostrils to access the tumor. This procedure carries a risk of severe postoperative complications due to the narrow approach through the nasal corridor, limiting the surgeon’s ability to navigate accurately and safely resect the tumor. Common complications include cerebrospinal fluid (CSF) leakage, meningitis, dysnatremia, vision impairment or carotid injury caused by damage to nearby blood vessels, nerves and the pituitary gland itself. To overcome these challenges, this work proposes a pipeline leveraging computer vision and artificial intelligence (AI) to reconstruct the patient’s anatomy from monocular endoscopic videos.

**Fig. 1:**
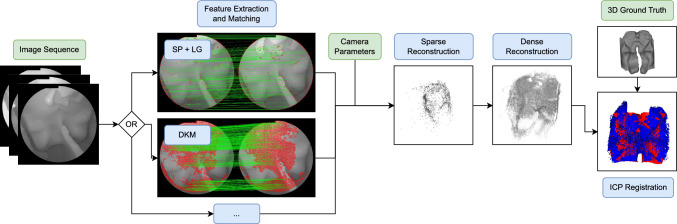
Overview of the reconstruction pipeline. For each image sequence, feature matches are extracted, sparsely reconstructed and densified using COLMAP, and evaluated against the ground-truth CAD model through ICP registration

Using a surgical dataset acquired on a high-fidelity phantom platform with textureless surfaces, we compare the performance of several learned feature extraction and matching methods—specifically, SuperPoint [2], DISK [14], ALIKED [15], SuperGlue [11], LightGlue [6] and Dense Kernelized Feature Matching (DKM) [4]—for Euclidean 3D reconstruction. These state-of-the-art feature detectors and matchers present potential solutions to overcoming the challenges faced by classical methods in textureless, monotonous scenes. As a first step toward augmented reality (AR) navigation, these 3D reconstructions could eventually be registered onto preoperative imaging data, such as MRI or CT scans, allowing for AI-assisted navigation to enhance the surgeon’s spatial awareness and overall safety of the procedure.

**Fig. 2:**
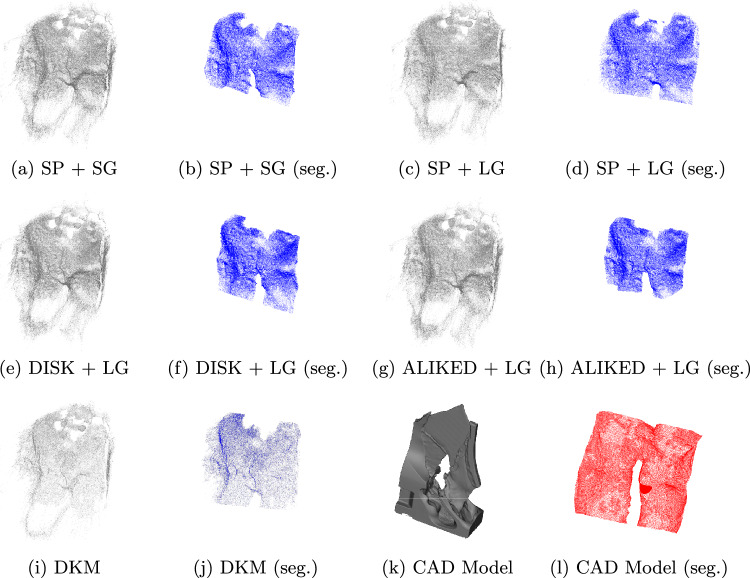
Reconstructed point clouds generated using COLMAP’s dense MVS reconstruction pipeline and their corresponding manually segmented surfaces (seg.) used for ICP registration

## Methods

The following is a brief overview of the proposed pipeline (Fig. 1) for 3D reconstruction of the sellar anatomy from 2D endoscopic videos. After the acquisition of multiple videos of simulated surgeries and calibration of the acquisition system, learned features were extracted and matched across video frames. Feature matches were then sparsely reconstructed and densified using COLMAP, and evaluated against the 3D ground truth through registration using the iterative closest point (ICP) algorithm.

### Dataset acquisition and camera calibration

Surgical video data were acquired as part of a user study involving 14 trainee surgeons, simulating the sellotomy (removal of the bony sellar floor) and tumor resection steps of pituitary surgery using a portable endoscope stack (STORZ TELE PACK +). The procedures were conducted on a high-fidelity phantom platform (UpSurgeOn TNSBox) featuring a 3D printed sellar bone. The 3D CAD model was generated from laser scans of the original part and used as ground truth. While anatomically correct, a critical aspect of the 3D printed phantoms is their highly uniform, textureless surfaces which are very different from those observed during real surgical procedures, where tissues and bone structures exhibit significantly more detail for feature extraction and matching. The recorded videos were preprocessed by extracting all frames, converting to gray scale, cropping and masking to remove pixels unrelated to the raw endoscope camera feed (pixels that were part of the acquisition system’s user interface). The intrinsic parameters of the endoscope’s camera were estimated using the *scikit-surgerycalibration* [13] and OpenCV [1] libraries on short sequences of a ChArUco calibration target scanned from multiple angles prior to each procedure.

### Feature extraction and matching

We evaluated several combinations of state-of-the-art learned feature extractors and matchers using their default parameters and weights, namely (1) SuperPoint with SuperGlue, (2) SuperPoint with LightGlue, (3) DISK with LightGlue and (4) ALIKED with LightGlue for sparse feature extraction and matching, and (5) DKM for dense feature extraction and matching, to highlight their distinct advantages in addressing accuracy, speed and robustness for 3D reconstruction of the phantom’s textureless surfaces. Short video sequences featuring exclusively the sellar bone structure and devoid of occlusions caused by instruments were selected to ensure consistency across trials. Feature extraction and matching were performed on all possible pairs of images and restricted to 8000 features per image.

### 3D reconstruction and evaluation

The generated feature matches and estimated camera intrinsic parameters were input into the COLMAP [12] library, which estimates both the camera poses (extrinsic parameters) and 3D position of matched features, to reconstruct the sellar anatomy in a two-stage process. Sparse point clouds were first generated using COLMAP’s sparse reconstruction pipeline on the set of all matched features. These reconstructions were then refined using COLMAP’s dense Multi-View Stereo (MVS) pipeline, which produces depth and normal maps for all registered images to generate dense point clouds and effectively filter out outliers. To evaluate the accuracy of the dense reconstructions, we employed the ICP algorithm as implemented in the Open3D library [16] to register against the ground-truth 3D CAD model.

## Results

### Experimental setup and metrics

For each of the 10 evaluated sequences, 50 images were uniformly extracted from the selected video segments at a rate of 3.33 Hz and processed through the reconstruction pipeline. The feature matches and sparse reconstructions were computed using an NVIDIA RTX 3090 GPU in all experiments. To evaluate the accuracy of the reconstructions and ensure consistent results across all trials, the ground-truth CAD model and reconstructed point clouds were manually segmented to remove all artifacts generated during the printing process that were not effectively part of the sellar bone (Fig. 2), and a manual preregistration (orientation and scale of the surfaces) was performed by establishing precise 3D-to-3D correspondences using distinctive geometrical structures (the surface peaks of the optic protuberances and carotids in each point cloud) to initialize the ICP algorithm and compute the RMSE. The average results on all 10 sequences were only performed using the two most accurate methods in the single sequence experiments (SuperPoint with LightGlue and DKM).

**Fig. 3:**
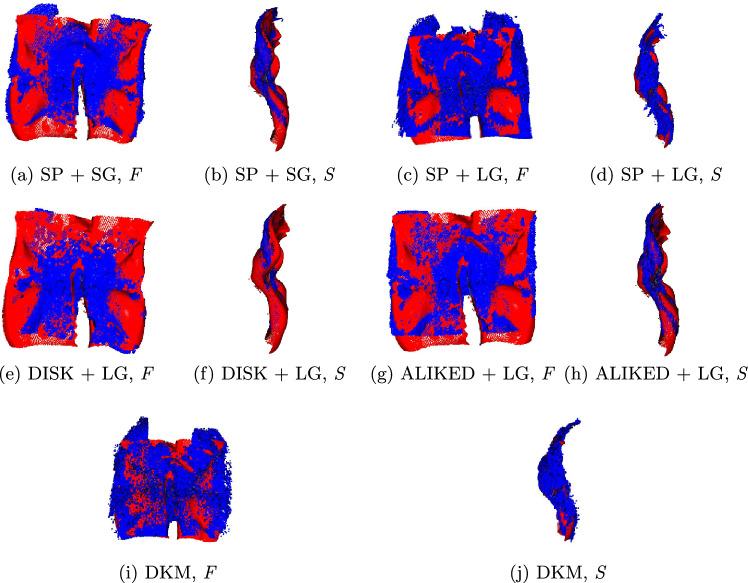
ICP registration visualizations. Ground-truth surface (in red) and densely reconstructed point clouds generated using COLMAP’s dense MVS reconstruction pipeline (in blue), both manually segmented. Below each figure, *F* and *S* denote the front and side views

**Table 1:** Reconstruction metrics on the best sequence. In the *Dense* columns, *Points* and *Points (ICP)* are the number of reconstructed points before and after segmentation for ICP registration

Method	Sparse reconstruction		Dense reconstruction		
	Points	Time (s)	Points	Points (ICP)	RMSE (mm)
SP + SG	4025	242.04	616925	426557	0.14
SP + LG	4086	**135**.**59**	616554	471530	0.13
DISK + LG	14110	1609.20	643391	463349	0.19
ALIKED + LG	13389	653.43	644462	435453	0.18
DKM	29964	1215.30	256620	158368	**0**.**11**

Bold values indicate the fastest method (Time) and best performing method (RMSE)

### Reconstruction performance

The ICP registrations of the densely reconstructed point clouds are displayed in Fig. 3, and all computed metrics are shown in Tables 1 and 2. Non-learned feature extractors, such as SIFT [7] and ORB [10], using a nearest neighbor matcher from the Kornia library [9], produced no valid reconstructions, as an insufficient number of features were detected in the images, resulting in no global matching across all images. These results were consequently not included in the tables. Regarding the accuracy of the evaluated methods, from a clinical perspective, the measured RMSEs are negligible (mostly inferior to a millimeter on average) and acceptable for visualization purposes. Although visually similar, we can observe in Fig. 3 that DKM more accurately captured the convexities of the ground-truth surface in detail, at 10 times the computation cost of the fastest method (SuperPoint with LightGlue). However, we also observe a three times increase in RMSE on average across all test sequences (Table 2) when compared to the best one (Table 1), pointing to limitations of the pipeline for sequences containing higher degrees of blur and low illumination, which hindered the performance of the feature extraction and matching stages. Comparing SuperGlue and LightGlue using SuperPoint features, we also noticed higher computation times and RMSE for SuperGlue, indicating the lower performance of this feature matcher on this sequence.

## Discussion and conclusion

In this work, we have compared several state-of-the-art, learned feature extraction and matching approaches for 3D reconstruction of the sellar anatomy during endoscopic pituitary surgery, addressing the challenge of reconstructing highly uniform and textureless surfaces. We observed that the DKM method outperforms all other feature extraction and matching methods for reconstruction accuracy, at the price of significantly higher computation times.

The two methods evaluated on all sequences show good performance, but the variability in reconstruction accuracy when comparing to the results obtained on the best sequence, as shown in Tables 1 and 2, can mainly be imputed to the frame extraction process. Sequences with blur or occlusions resulted in sparser, geometrically inaccurate reconstructions, whereas sequences with consistently clear views of the sella produced denser, less noisy reconstructions. A keyframe sampling criterion [3], only extracting high-quality frames, could be used in future works to increase consistency.

**Table 2:** Average reconstruction metrics on 10 sequences for the two best-performing methods

Method	Sparse reconstruction	Dense reconstruction	
	Time (s)	Points	RMSE (mm)
SP + LG	**150**.**26**	247606	0.41 ± 0.33
DKM	996.18	131883	**0**.**33** ± 0.23

Bold values indicate the fastest method (Time) and best performing method (RMSE)

As a first step toward AR-assisted navigation, an extension of this work could investigate registering the reconstructed surface to a preoperative scan of the patient’s anatomy. A practical solution would require a two-stage implementation, in which the surface is first reconstructed and registered, before estimating the pose of the endoscope in real time using matched 2D-to-3D correspondences and a lightweight PnP algorithm [5]. The speed of the two best-performing approaches was evaluated for pair-wise image matching with a limited number of features per image. SuperPoint and LightGlue computation time was 0.19 s, while DKM was 1.24 s, indicating the potential of these methods for real-time pose estimation, and more generally, for AR-assisted navigation in endoscopic pituitary surgery.

## References

[1] Bradski G (2000) The OpenCV Library. Dr. Dobb’s J Softw Tools

[2] DeTone D, Malisiewicz T, Rabinovich A (2018) Superpoint: self-supervised interest point detection and description. In: Proceedings of the IEEE conference on computer vision and pattern recognition (CVPR) workshops

[3] Dias NJB, Laureano GT, Da Costa RM (2023) Keyframe selection for visual localization and mapping tasks: a systematic literature review. Robotics. 10.3390/robotics12030088

[4] Edstedt J, Athanasiadis I, Wadenbäck M, Felsberg M (2023) Dkm: dense kernelized feature matching for geometry estimation. In: Proceedings of the IEEE/CVF Conference on computer vision and pattern recognition (CVPR), pp 17765–17775

[5] Lepetit V, Moreno-Noguer F, Fua P (2009) EPnP: an accurate o(n) solution to the PnP problem. Int J Comput Vision 81(2):155–166

[6] Lindenberger P, Sarlin PE, Pollefeys M (2023) Lightglue: local feature matching at light speed. In: Proceedings of the IEEE/CVF International Conference on Computer Vision (ICCV), pp 17627–17638

[7] Lowe DG (2004) Distinctive image features from scale-invariant keypoints. Int J Comput Vis 60(2):91–110

[8] Marcus HJ, Khan DZ, Borg A, Buchfelder M, Cetas JS, Collins JW, Dorward NL, Fleseriu M, Gurnell M, Javadpour M, Jones PS, Koh CH, Layard Horsfall H, Mamelak AN, Mortini P, Muirhead W, Oyesiku NM, Schwartz TH, Sinha S, Stoyanov D, Syro LV, Tsermoulas G, Williams A, Winder MJ, Zada G, Laws ER (2021) Pituitary society expert delphi consensus: operative workflow in endoscopic transsphenoidal pituitary adenoma resection. Pituitary 24(6):839–853 34231079 10.1007/s11102-021-01162-3 PMC8259776

[9] Riba E, Mishkin D, Ponsa D, Rublee E, Bradski G (2020) Kornia: an open source differentiable computer vision library for pytorch. In: Proceedings of the IEEE/CVF winter conference on applications of computer vision (WACV)

[10] Rublee E, Rabaud V, Konolige K, Bradski G (2011) Orb: An efficient alternative to sift or surf. In: 2011 International conference on computer vision, pp 2564–2571

[11] Sarlin PE, DeTone D, Malisiewicz T, Rabinovich A (2020) Superglue: learning feature matching with graph neural networks. In: Proceedings of the IEEE/CVF conference on computer vision and pattern recognition (CVPR)

[12] Schönberger JL, Zheng E, Frahm JM, Pollefeys M (2016) Pixelwise view selection for unstructured Multi-View stereo. In: Computer Vision – ECCV 2016, Springer International Publishing, pp 501–518

[13] Thompson S, Dowrick T, Ahmad M, Xiao G, Koo B, Bonmati E, Kahl K, Clarkson MJ (2020) SciKit-Surgery: compact libraries for surgical navigation. Int J Comput Assist Radiol Surg 15(7):1075–1084 32436132 10.1007/s11548-020-02180-5 PMC7316849

[14] Tyszkiewicz M, Fua P, Trulls E (2020) Disk: Learning local features with policy gradient. In: Larochelle H, Ranzato M, Hadsell R, Balcan M, Lin H (eds) Advances in neural information processing systems, vol 33. Curran Associates Inc, pp 14254–14265

[15] Zhao X, Wu X, Chen W, Chen PCY, Xu Q, Li Z (2023) Aliked: a lighter keypoint and descriptor extraction network via deformable transformation. IEEE Trans Instrum Meas 72:1–16. 10.1109/TIM.2023.327100037323850

[16] Zhou QY, Park J, Koltun V (2018) Open3d: A modern library for 3d data processing

